# Using human induced pluripotent stem cell-derived cardiomyocytes to understand the mechanisms driving cardiomyocyte maturation

**DOI:** 10.3389/fcvm.2022.967659

**Published:** 2022-08-12

**Authors:** Homa Hamledari, Parisa Asghari, Farah Jayousi, Alejandro Aguirre, Yasaman Maaref, Tiffany Barszczewski, Terri Ser, Edwin Moore, Wyeth Wasserman, Ramon Klein Geltink, Sheila Teves, Glen F. Tibbits

**Affiliations:** ^1^Department of Biomedical Physiology and Kinesiology, Simon Fraser University, Burnaby, BC, Canada; ^2^Department of Molecular Biology and Biochemistry, Simon Fraser University, Burnaby, BC, Canada; ^3^Cellular and Regenerative Medicine Centre, BC Children’s Hospital Research Institute, Vancouver, BC, Canada; ^4^Department of Cellular and Physiological Sciences, University of British Colombia, Vancouver, BC, Canada; ^5^Department of Medical Genetics, University of British Colombia, Vancouver, BC, Canada; ^6^BC Children’s Hospital Research Institute, Vancouver, BC, Canada; ^7^Department of Pathology and Laboratory Medicine, University of British Colombia, Vancouver, BC, Canada; ^8^Department of Biochemistry and Molecular Biology, University of British Colombia, Vancouver, BC, Canada; ^9^School of Biomedical Engineering, University of British Columbia, Vancouver, BC, Canada

**Keywords:** transcriptional regulation, cell signaling, preterm heart, hiPSC-derived cardiomyocyte, mTOR pathway

## Abstract

Cardiovascular diseases are the leading cause of mortality and reduced quality of life globally. Human induced pluripotent stem cell-derived cardiomyocytes (hiPSC-CMs) provide a personalized platform to study inherited heart diseases, drug-induced cardiac toxicity, and cardiac regenerative therapy. However, the immaturity of CMs obtained by current strategies is a major hurdle in utilizing hiPSC-CMs at their fullest potential. Here, the major findings and limitations of current maturation methodologies to enhance the utility of hiPSC-CMs in the battle against a major source of morbidity and mortality are reviewed. The most recent knowledge of the potential signaling pathways involved in the transition of fetal to adult CMs are assimilated. In particular, we take a deeper look on role of nutrient sensing signaling pathways and the potential role of cap-independent translation mediated by the modulation of mTOR pathway in the regulation of cardiac gap junctions and other yet to be identified aspects of CM maturation. Moreover, a relatively unexplored perspective on how our knowledge on the effects of preterm birth on cardiovascular development can be actually utilized to enhance the current understanding of CM maturation is examined. Furthermore, the interaction between the evolving neonatal human heart and brown adipose tissue as the major source of neonatal thermogenesis and its endocrine function on CM development is another discussed topic which is worthy of future investigation. Finally, the current knowledge regarding transcriptional mediators of CM maturation is still limited. The recent studies have produced the groundwork to better understand CM maturation in terms of providing some of the key factors involved in maturation and development of metrics for assessment of maturation which proves essential for future studies on *in vitro* PSC-CMs maturation.

## Introduction

Cardiovascular diseases (CVD) are the leading cause of mortality, reduced quality of life, and the associated burden on healthcare systems (1) globally. Despite significant advances in the development of CVD therapeutics, about 90% of promising drugs based on animal models show poor outcomes in human clinical trials (2). These models are practical for laboratory research purposes, but the physiological differences between animal models and humans ultimately limits their potential to provide clear insight into CVD mechanisms and therapeutics (3). For example, certain inherited arrhythmias and cardiomyopathies are challenging to study in animal models because the underlying disease-causing genetic mutations cause severe phenotypic effects that often result in sudden cardiac death in animals (4). Human genetic backgrounds can also determine an individual’s response to CVD treatments and their side effects which needs to be studied using an individualized research platform (5).

Advancements in the field of cardiovascular regenerative medicine through the use of human induced pluripotent stem cell-derived cardiomyocytes (hiPSC-CMs) and other state-of-the-art techniques, including genome editing, have made it possible to better understand the molecular mechanisms underlying developmental abnormalities involved in congenital heart diseases, drug- induced cardiac toxicity, and inherited cardiomyopathies (6). Furthermore, generating hiPSC-CMs requires minimally invasive techniques to collect human blood or skin cells, which are then reprogrammed into pluripotent stem cells (hiPSCs) through expression of key transcription factors (TFs) Sox2, Oct4, Klf4, c-Myc, followed by differentiation into CMs (hiPSC-CMs) (7).

However, a major barrier to the clinical relevance of hiPSC-CMs is the immaturity of CMs obtained by current differentiation protocols. The same issue with immaturity is also observed with the induced-cardiomyocytes (iCMs) that have been transdifferentiated from fibroblasts by overexpression of specific TFs such as Gata4, Mef2c, Tbx5, MYOCD, and others (8, 9). Hence, a major focus of current research is to gain insight into the mechanisms directing CMs to a fully matured state in order to improve the maturation-inducing strategies.

Immature hiPSC-CMs differ from adult CMs over a broad spectrum of parameters that includes but is not limited to underdeveloped mitochondria (both in amount and with cristae structure), limited fatty acid oxidation capacity, lack of T-tubules, disorganized myofibrils, and sarcomeres, and altered transcriptional/post-transcriptional networks that generate electrical/mechanical forces and excitation-contraction coupling. Researchers have used various approaches including electrical/mechanical/chemical stimulation, extended culture duration, heart tissue engineering, various ECM components, non-coding-RNAs, and co-culturing with fibroblasts/human Mesenchymal Stem Cells to induce maturation, but these approaches are only partially effective (10).

In this review, we delve deeper into various aspects of CM maturation, summarize recent findings on understanding mechanisms in maturation of hiPSC-CMs, and transcriptional regulation critical in directing hiPSC-CMs maturation.

## Insight into cardiomyocyte maturation derived from the prenatal to postnatal transition

**FIGURE 1:**
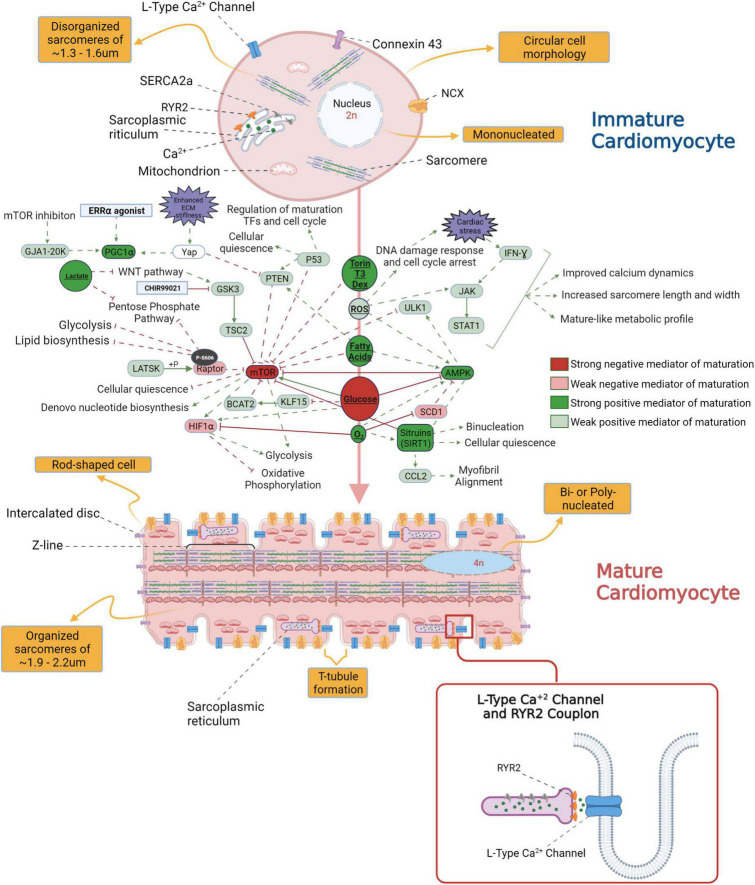
An overview of the morphological differences between immature and mature cardiomyocytes (CM) and the signaling pathways potentially involved in the transition from fetal to adult CM stage.

Numerous factors initiate the *in vivo* CM maturation during gestation and post – parturition; these include the changes in access to oxygen, shift in CM fuel metabolism from glucose to free fatty acids upon nursing of neonates with breast milk, and increased demand for ventricular pressure development (11). In the prenatal stage of cardiac development and a few months into the post-natal stages, hyperplasia drives the development of the heart’s cellular structure. In the first decade of human life, the growth of the heart occurs mainly by hypertrophy or by increasing the size of the individual CMs as they are now in a post mitotic state (12). Communication between individual cardiac myocytes through well-developed adhesive and gap junctions creates an electromechanical and functional syncytium through myocardial maturation. Organized and well-developed T-tubules, dyadic distribution and structure, and functional calcium release units are critical components of mature CMs in the adult heart. For a detailed overview on the characteristics of adult versus immature CMs refer to Figure 1.

Knowledge of maturation inducing factors (MIFs) is rapidly expanding but most of this information is phenomenological in nature and not mechanistic. In the following section, we will provide an overview of current knowledge on CM maturation with inspiration from major MIFs involved in prenatal to postnatal transition.

## Signaling pathways potentially associated with human induced pluripotent stem cell-derived cardiomyocytes maturation

### Oxygen sensing pathways

The transition to an oxygen enriched environment has been shown to improve CM maturation by cell cycle arrest through the induction of DNA damage response (13). One study, albeit in hyperoxia conditions, indicated the loss of CM proliferation due to suppressed expression of the *de novo* fatty acid synthesis genes *Fasn* and *Scd1* in the left atria, but not ventricle, of mice and HL-1 atrial cell line. Scd1 was also implicated in left atrial tissue obtained from stillborn infants under hyperoxia. hyperoxia activated the fatty acid metabolism genes *PPARGC1A* involved in mitochondrial maturation, *HADHA* involved in long chain fatty acid β oxidation, and *LPL* lipoprotein lipase, all of which are involved in metabolic aspects of CM maturation. The loss of Scd1 was previously linked to increased production of reactive oxygen species (ROS) and reduced proliferative potential of neonatal CM which aligns with DNA damage response. Moreover, loss of Scd1 activates AMP-activated protein kinase (AMPK) which can negatively regulate mTORC1 signaling (4).

The impact of hypoxia and Hypoxia-Inducible Factors (HIFs) on interfering with aspects of CM maturation has been previously investigated. Hu et al. showed improvement in metabolic and contractile maturation of hiPSC-CMs using small molecule inhibition of HIF-1α to counteract its increased activity under glucose enriched culture medium (14). Similarly, HIF-1α inhibitor in combination with peroxisome proliferator activated receptor α (PPARα) was shown to better enhance CM maturation (15). Transcriptome analysis of embryonic to adult mouse hearts shows the activity of HIF-1α from mid to late stages of gestation (16). HIF-1α activity positively regulates normal CM proliferation and glycolysis in fetal CMs, and its activity is posited to be essential in CM development. Menendez-Montes et al. recently showed that the loss of HIF-1α signaling in mouse Nkx2.5 cardiac progenitors does not interfere with normal cardiac development and adult heart function. Loss of *Hif1a* resulted in decreased mRNA and protein expression of glycolysis enzymes, increased mitochondrial number, and alternative amino acid catabolism as the source of energy without affecting CM proliferation (17).

### Nutrient sensing pathways

A major step in CM maturation is the transition from glucose utilization by glycolysis to fatty acid oxidation and increased mitochondrial respiration upon nursing after birth. In this regard, high glucose levels have been shown to negatively affect CM maturation. For instance, Nakano et al. demonstrated that glucose interferes with hESC-CM maturation through the induction of pentose phosphate pathway (PPP) mediated nucleotide biosynthesis (18). Moreover, glucose inhibits the expression of the TF Krüppel Like Factor 15 (KLF15), which regulates the branched chain amino acid (BCAA) metabolism (19). A recent proteomics study on the mitochondria of adult/neonatal mouse heart and hiPSC-CMs (days 35, 75, 120), detected KLF15 as a major upstream regulator of CM maturation with enhanced activity during CM differentiation (20). KLF15 physically interacts with PPARa and its silencing has been shown to decrease CM response to PPARa agonists (i.e., ones typically used in CM chemical maturation protocols) (21). KLF15 was recognized to be a direct target of the glucocorticoid receptor in skeletal muscle and it has been shown to induce the expression of BCAT2 (BCAA transaminase 2) which degrades BCAAs and subsequently leads to the inhibition of mTOR (22). The single cell RNAseq analysis on short term (i.e., 3 days in duration starting at day 10 of differentiation) of glucose starved hiPSC-CMs also determined an enhancement in the CM purity and expression of maturation related genes (23).

**FIGURE 2:**
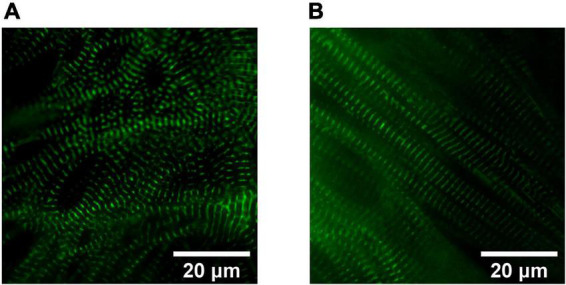
Sarcomere organization in hiPSC-derived cardiomyocytes (CMs). Widefield fluorescence images (Videos of beating CMs available) of 1 Hz paced hiPSC-derived CMs expressing mEGFP-labeled α-actinin-2 at sarcomeric z-disks (cell line ID: AICS-0075 cl.85). **(A)** Human iPSC-CMs cultured for 40 days in RPMI1640/B27 have less sarcomeric organization compared to **(B)** Day 40 human induced pluripotent stem cell-derived cardiomyocytes (hiPSC-CMs) treated for 4 weeks in a metabolic maturation media (MM).

**FIGURE 3:**
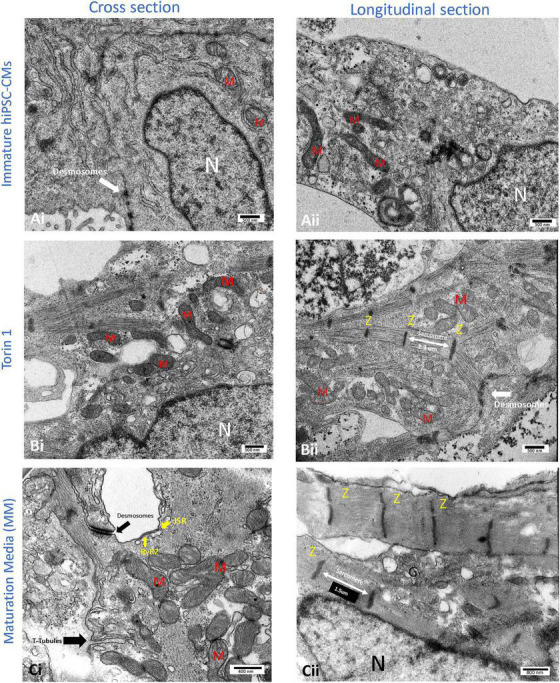
Transmission electron microscopy (TEM) images of the cellular structure of human-induced pluripotent stem cell-derived cardiomyocytes (hiPSC-CMs) after 14 days of 200 nM Torin1 (mTORC1/C2 inhibitor) exposure **(B)** or 4–5 weeks of metabolic maturation media (MM) **(C)**. In comparison to untreated hiPSC-CMs **(A)**, cells treated with Torin 1 and MM media show dramatically enhanced ultrastructure. In cells treated with Torin 1, prominent Z-lines with aligned and elongated sarcomeres (∼1.3 μm) are observed **(Bii)**. In addition to desmosomes, golgi, and endoplasmic reticulum, the formation of interspersed mitochondria is also observed **(Bi)**. In cells incubated with MM media for four to five weeks, packed mitochondria with well-formed cristae, more significant junctional SR, with possible T-tubule formation are also observed **(Ci)**. In addition to dense peri-nuclear mitochondria, surface dyads with RyR2 on junctional SR were observed **(Ci)**. JSR, junctional SR. M, mitochondrion. N, nucleus. RyR2, type II ryanodine receptor. Z, Z line. i, cross sections of the hiPSC-CM. ii, longitudinal section of the hiPSC-CM.

With respect to the impact of fuel source on CM maturation, Feyen et al. developed a Metabolic Maturation media (MM) consisting of 3 mM glucose and high levels of albumin-bound fatty acids (AlbuMAX) among other factors. After metabolic purification of beating hiPSC-CMs with sodium L-lactate in media without glucose, MM treatment was performed for 5 weeks; subsequently, functional analysis revealed a promising enhancement in the maturation of FFA metabolism, electrophysiology, structure, Ca^2+^ handling, and contractility (24). RNAseq analysis also demonstrated the upregulation of genes involved in mitochondrial metabolism, cristae formation, calcium handling, sarcomeric components and ion channels including *KCNJ2* which encodes Kir2.1 ion channels (24). For more information on the impact of MM on CM structural maturation and sarcomeric alignment, see Figures 2, 3. The transient exposure to lactate as part of metabolic purification of hPSC-CMs was recently shown to induce a long-term transcriptomic shift in the expression of genes involved in the maturation of calcium handling, contractility, and decreased proliferation markers as well as Wnt signaling and PPP (25).

In a recent study, supplementation of day 15 hiPSC-CMs growth media with 10 mM galactose, 100 μM oleic acid, and 50 μM palmitic acid for 20 days demonstrated a significant enhancement in ultrastructure, Ca^2+^ transients, contractile performance, and action potential kinetics. Moreover, transcriptomic analysis using microarrays indicated an upregulation of maturation genes with increased hallmarks of PTEN, PPARα, PPARα/RXR signaling, and a decrease in mTOR signaling (26). In another study, 105 μM palmitate-albumin, 81 μM oleic acid-albumin, and 45 μM linoleic acid-albumin complexes were used to treat day ∼20 hPSC-CMs for 2 weeks. The results of which showed similar enhancements in structural and functional maturation of hPSC-CMs. The activities of AMPK, ERK, and p38-MAPK were upregulated as evidenced by western blot analysis (27).

In a study by Sarikhani et al., AMPK signaling was shown to play a key role in CM differentiation. The dynamics of AMPK signaling in hiPSC-CMs was shown to be a hyperphosphorylation state on day 9–11 of differentiation toward a hypo phosphorylation state at later stages (28). As a follow-up, treatment of hiPSC-CMs with 1 mM AICAR (an AMP analog and AMPK agonist) for a limited time span of 5 days from day 9-14 of differentiation enhanced the expression of sarcomeric proteins encoded by *TNNI3, TNNT2*, CD36/GLUT4 involved in fatty acid/glucose uptake, and increased mitochondrial respiration as shown by a Seahorse assay. AMPK activation, however, decreased the expression of *KCNJ2* (Kir2.1) which encodes the inward rectifier K^+^ channel (I_*K*1_), a commonly used maturation index. The consequences of AMPK activation on CM electrophysiology and structural/functional maturation were not addressed in this study. The results of this study also demonstrated the role of sirtuin proteins (with histone deacetylase activity) in mediating the effects of AMPK on enhanced cardiogenesis through change in the chromatin accessibility of sarcomeric genes and cardiac TFs involved in mid-late CM differentiation stage (*GATA4, NKX2-5*) (28).

Ye et al., observed an increased degree of phosphorylated AMPK in hiPSC-CMs from day 10 to day 30 of differentiation. At day 23, they further treated the hiPSC-CMs with 0.5 mM AICAR for 7 days and performed functional analyses at day 30. The results of this study demonstrate a significant enhancement in CM adult-like morphology and mitochondrial maturation as shown by Seahorse assay and transmission electron microscopy (TEM). Moreover, gene expression analysis for genes previously implicated in CM maturation were identified the upregulation of sarcomeric genes and voltage-gated ion channels encoding genes (*SCN5A, CACNA1C, KCNE1, KCNJ2, KCND2, KCNQ1, KCNH2*) among others. *KCNJ2* (I_*K*1_) expression was induced by AICAR in contrast to the previous study. However, functional consequences though the electrophysiological assessment was not performed (29). AMPK is also shown to promotes cell cycle arrest in mouse embryonic fibroblasts (MEFs) by phosphorylating p53 at Ser 15 in glucose deprived conditions (30) but its potential relevance to the regulation of hiPSC-CMs maturation in low glucose media needs to be determined.

A recent study using a murine model, demonstrated the role of AMPK in the regulation of CM contractility and shape in addition to its role in the regulation of cellular metabolism. The upregulation of phosphorylated AMPK from 8 weeks after birth and the localization of AMPK to the intercalated disks (IDs; i.e., structural entities connecting adjacent CMs) were demonstrated to be mediated by CM contractility-induced trafficking of AMPK to IDs. This observation was simultaneous with the formation of well-organized IDs around weeks 7–8 after birth (31). The kinase activity of AMPK at IDs modulates the dynamics of microtubules as regulators of intracellular stiffness thereby affecting CM shape (i.e., length/width ratio) (31). The role of AMPK activity, subcellular localization, and regulation of microtubule dynamics as part of hPSC-CM morphological and functional maturation in response to different mechanotransduction signals [e.g., extracellular matrix (ECM) stiffness and force of contraction] is an interesting area which requires further investigation.

Sirtuin proteins have also been linked to CM maturation in some studies. Shin et al. showed the essential role of Silent mating type information regulation 2 homolog 1 (SIRT1) protein in the induction of CM binucleation in the postnatal rat heart and H9c2 cells lines which are derived from embryonic rat CMs. Moreover, hypoxia was demonstrated to suppress the mRNA and protein expression of SIRT1 (32). The Sirtuin proteins, including SIRT1, are also shown to modulate cellular quiescent state which is critical for CM maturation (33, 34). The role of Sirtuins therefore, needs to be investigated further with respect to this aspect of CM maturation (35). In particular, the peak of SIRT1 expression is observed from the perinatal to neonatal stages of mouse CM development (36). A very recent study by Fang et al. suggests a crucial role of SIRT1 in the proper formation of aligned myofibrils partly through transcriptional regulation and induction of CCL2 chemokine signaling pathway. SIRT1 deficiency results in disturbed CMs/myofibrils orientation and alignment in both *in vitro* hESC-CMs and *in vivo* mouse heart (37).

Another recent study examined a relevant aspect of prenatal to postnatal nutrient utilization on CM maturation. Yang et al. aimed to mimic the effects of starvation on hESC-CMs maturation which was inspired by the enhanced CM maturation due to sudden interruption of nutrient supply upon birth in the neonatal heart. This short starvation period induces autophagy in the heart which is believed to have an essential role on cardiac functional maturation. The authors used Earles’ Balanced Salt Solution (EBSS), starvation- induced autophagy, for an optimized timeframe of 2 h daily EBSS treatment for 10 days starting at day 15 of differentiation (or before day 20–30 in which maturation arrest may happen). The result was an increase in CM size, sarcomere length, and the percent of multinucleated CMs. Moreover, the mitochondrial maturation was increased as shown by enhanced mitochondrial biogenesis (MitoTracker staining), mitochondrial activity (mitochondrial membrane potential indicator, JC1), basal/maximal respiration, and ATP production (Seahorse assay). Electrophysiological maturation and improved calcium handling were also shown using patch-clamp and calcium imaging (Fluo-4 calcium indicator). However, there were no significant changes in the glycolysis rate, CMs resting membrane potential, and maximum upstroke velocity compared to control CMs (38).

The pro-maturation effects observed in the Yang et al. study were hypothesized to be through AMPK and SIRT1 which regulate CM autophagy and also act as negative regulators of mTORC1 (38). This is in accordance with what was discussed earlier on the role of nutrient sensing pathway in CM maturation. The regulation of autophagy by mTORC1 has been well documented. mTORC1 not only inhibits autophagy induction by phosphorylation of ULK1, it also inhibits the elongation and maturation of the autophagosome complex (39). Deptor, negative regulator of mTOR kinase activity, is shown to accumulate upon glucose deprivation and upregulates the LC3-II protein expression/decrease p62 protein, both of which were observed during starvation-induced autophagy (40, 41). The possible connection between the positive effects of low glucose levels on CMs maturation and autophagy should be investigated in future studies. Dexamethasone (DEX) has been shown to enhance mouse ESC-CMs maturation through the induction of mitophagy (i.e., selective degradation of mitochondria through autophagy). The authors show that mitophagy induction through the parkin protein is responsible for enhanced CM maturation. The inhibition of mitophagy and parkin protein expression both abrogated the maturation effects of DEX (42).

### mTOR Regulation

A common aspect shared between many of the MIFs is their association with the nutrient sensing mTOR (mechanistic Target of Rapamycin) pathway. mTOR is a kinase acting as the core constituent of mTORC1 and mTORC2 complexes that belongs to the PI3K-related kinase (PIKK) family (43). The mTOR pathway is evolutionarily conserved across species; mTOR signaling is documented to be as a major contributor in coordination of timing between growth and maturation at the end of larval stage in Drosophila through affecting metabolic pathways (44). The involvement of mTOR in CM development has been the subject of recent research. Specifically, inhibition of mTORC1 by Rapamycin in human cardiac progenitor cells reduces replicative senescence and enhances CM differentiation, both critical components of the maturation process (33). The inhibition of mTORC2 by RICTOR knockdown (part of mTORC2 complex), however, was shown to interfere with mouse ESC-CMs differentiation, in particular ventricular CMs. mTORC2 inhibition also induced abnormal electrophysiology and altered distribution of connexin 43 (CX43), N-Cadherin and desmoplakins at cell-cell junctions (45). On the other hand, a recent proteomics study on the mitochondria of hiPSC-CMs at different timepoints of differentiation and from neonatal/adult mouse heart, identified RICTOR as an upstream regulator of CM maturation whose expression is inactivated at later time points and in adult mouse heart (20). Further studies are needed to investigate the mechanisms by which mTORC2 regulates CM differentiation and whether its activity is modulated in a stage-specific and at subcellular level during CM development.

Garbern et al. recently showed that mTOR activity determines whether CMs transit toward senescent or quiescent states. mTORC1 was previously implicated as being a key factor modulating cellular transition from alert quiescence to deep quiescence, which is a state in which cells are not replicative but are both metabolically and transcriptionally active (34). In this study hiPSC-CMs were treated with 200 nM Torin 1 (mTORC1/C2 inhibitor) starting from 2 days after the onset of beating (day 9) for 7 days. Although the results of functional studies and targeted gene expression analysis showed a significant enhancement in different aspects of CM maturation, the expression of some key genes including specific ion channel encoding genes were still far from that of the adult heart (34). Upregulation of p53 together with inhibition of mTOR was demonstrated to induce quiescent state that improves the maturation of hiPSC-CMs (34). As such, the authors argue that mTOR regulates CM maturation through p53-induced regulation of cardiac TFs (34).

The crosstalk between mTOR and p53 has been extensively studied in other cell types. Many negative regulators of mTOR (e.g., TSC2, REDD1, AMPK6, PTEN, Sestrin1/2, Deptor) are transcriptional targets of p53. Moreover, p53 regulates the expression of miR-100, -101, -145, -155, -199a-3p; all of which are involved in regulation of mTOR pathway. On the other hand, mTOR signaling can repress or activate p53 depending on cell type and stress conditions (46). The mTOR and p53 crosstalk, therefore, need to be investigated in CMs development. The role of p53 in cardiac transcriptional regulation was previously studied in an *in vivo* study of adult mouse heart. It was shown that p53 regulates the transcription of genes involved in mitochondrial bioenergetics, glucose/fatty acids metabolism, and can form complexes with other TFs involved in CM development such as GATA4, ESRRγ, and MEF2A. p53 was also shown to directly bind to the promoters of PGC-1α, CPT1b, and P21 (47).

Increased PI3K/AKT/mTOR signaling has been observed in heart failure. The activation of CM fetal gene programs and alteration of substructure to immature in heart failure have been previously documented (48–51). Moreover, one of the hallmarks of metabolic reprogramming in human cardiomyopathies and mouse failing heart is the accumulation of BCAA due to defects in catabolizing BCAA (52). Glucose-mediated BCCA accumulation has been shown to be crucial in the activation of mTOR-induced hypertrophic growth in CMs (19). The activation of mTOR by BCCA has been previously linked to increased ROS production and mitochondrial dysfunction in other cell types such as peripheral blood mononuclear cells (PBMCs) (53). Nucleotide deprivation is suggested to be critical in CM maturation as previously mentioned (18). mTOR signaling is also responsible for the regulation of *de novo* nucleotides biosynthesis and PPP (54, 55). It remains to be investigated whether and by which mechanisms mTOR signaling might contribute to the inhibitory effects of glucose on CM maturation. Interestingly, the detailed mechanisms by which nucleotide deprivation affects CM maturation has not been studied. However, previous research has shown the critical role of mTORC1 in mediating the balance between nucleotide synthesis and demand. According to a study by Valvezan et al. in mouse TSC2 (upstream negative regulator of mTOR activity) deficient tumor cells, the pharmacological inhibition of *de novo* biosynthesis of guanine nucleotides by Mizoribine caused a depletion of dNTP sources. The constitutive ribosome biosynthesis by active mTORC1 coupled with Nucleotide deprivation (due to Mizoribine) was shown to cause replication stress and DNA damage response through anabolic imbalance (55). Thus, it would be interesting to further evaluate if this metabolic vulnerability can also be modulated to enhance CM maturation as suggested by previous research (18).

According to a recent study by Miklas et al., amino acid-induced activation of mTOR signaling is required for zebrafish heart regeneration. Moreover, CM cell cycle reentry and proliferation (suppressed as part of CM maturation) in the first week of neonatal mouse heart development and *in vitro* hESC-CMs were also shown to be dependent on activation of Wnt/B-catenin and mTORC1 signaling (56). In fact, Wnt/B-catenin activates the mTOR pathway through inhibition of GSK3, an activator of the mTOR upstream inhibitor TSC2. CHIR99021 which is typically used on day 0 of CM differentiation, inhibits GSK3β highlighting the possible role of mTOR pathway in early cardiogenesis (56, 57). Interestingly, GSK3β inhibition by CHIR99021 treatment is demonstrated to accelerate hiPSC-CM expansion and interfere with CM maturation as shown by the decreased expression of genes involved in various aspects of CM maturation (58).

Another pathway recognized as a highly conserved major regulator of heart size and growth is Hippo pathway. The cross-talk between Hippo and mTOR signaling was previously documented and it is shown to be mediated via Yes associated protein (Yap) as the major downstream target of Hippo pathway (59). Yap is shown to activate mTORC1/2 in human MCF10A breast cell line by induction of miR-29 mediated inhibition of PTEN (negative regulator of PI3K activity, thereby impacting downstream mTOR signaling) (60). Moreover, the positive feedback loop between mTOR-Yap, nucleus co-localization, and direct protein interaction is reported by a previous study in cancer cells (61). Additionally, the Hippo pathway LATS kinase was recently discovered to phosphorylate Raptor (part of mTORC1 complex) at Serin 606 thereby inhibiting mTORC1 activity. The Raptor S606 phosphorylation negatively affected glycolysis, PPP, and lipid biosynthesis (62); all of which are known to be suppressed during CM maturation. As such, it remains to be investigated whether Hippo-mTOR pathway crosstalk is also observed in CMs and whether this regulatory mechanism is involved in the hiPSC-CM transition through fetal to adult stage. Interestingly, Yap has recently been recognized to be the major target mediating the CM maturation promoting effects of peroxisome proliferator-activated receptor (PPAR) coactivator-1 (PGC1) signaling (63). While Yap is expected to interfere with CM maturation due to its proliferation enhancing effects, it also has a crucial role in the induction of hypertrophic response which is part of normal CM growth. Therefore, there may be a stage-specific necessity for Yap activity in the normal CM maturation of functional and structural elements.

One factor that likely interferes with full electrophysiological maturation of hiPSC-CMs, is the lack of proper localization of connexin 43 (CX43) proteins within the IDs. The connexin 43 encoding gene (i.e., *GJA1*) is subjected to alternative translation producing multiple n-terminal truncated isoforms observed in the human heart (e.g., GJA1-43K (full length), GJA1-32k, -29k, -26k, -20k). Inhibition of mTORC1 signaling and thereby Cap-dependent translation, modulates Internal Ribosome Entry Sites (IRES)-mediated translation and generation of smaller CX43 isoforms. The smaller isoforms of CX43 have been shown to regulate CX43 trafficking. GJA1-20K, dominantly expressed in human heart compared to other isoforms, mediates the trafficking of CX43 to the cell membranes at gap junctions (64). Basheer et al. showed that GJA1-20K regulates this process by interacting with actin filaments and microtubules by stabilizing the filamentous actin and further targeting of CX43 vesicles along microtubules to the cell surface (65).

GJA1-20K has been also implicated in microtubule-based transport of mitochondria to the cell periphery and protection of mitochondria from oxidative stress under stressed conditions (66). Under ischemia/reperfusion (I/R) injury in mouse heart, upregulated GJA1-20K expression induces mitochondrial biogenesis and decreases mitochondrial respiration to counteract oxidative phosphorylation (67). The role of smaller CX43 isoforms has not been studied in hiPSC-CM maturation and it remains to be evaluated whether these isoforms regulate the trafficking of CX43 to IDs and the increased oxidative stress as part of metabolic maturation of *in vitro* hiPSC-CMS. In particular, GJA1-20K upregulation was shown to induce the expression of PGC1-1α which is recognized to have a critical role in CM maturation (63, 67, 68). Other CX43 isoforms such as GJA1-11k, albeit not studied in CMs for its expression or function, has been shown to suppress cell cycle progression from the G0/G1 phase to the S phase regulating cell proliferation (69). Interestingly, Zeitz et al. showed that GJA1 5′ UTR containing IRES elements is shortened due to aging in mouse heart, under hypoxic stress, and TGF-beta exposure in hiPSC-CMs among other tested cells. The shortening of GJA1 5’ UTR limited alternative translation of GJA1-20K and resulted in reduced formation of gap junctions in mouse heart (70). Other than CX43, other genes being identified to undergo alternative translation include P53 (p53/47) (71) and human glucocorticoid receptor (72) among others. Interestingly, p53 activation (via Nutlin 3a) along with Torin inhibition of mTOR kinase activity was shown to better enhance CM maturation by Garben et al. (34). It may be interesting to evaluate whether p53 isoforms generated by cap-independent translation due to suppressed mTOR activity are contributing to enhance CM maturation at the proteome level. As such, it would be a promising endeavor to further distinguish how cap-dependent and cap-independent translation modulated by change in mTOR signaling affect CM proteome toward early and late stages of CM differentiation. This will further produce a reference for the evaluation of the success of current PSC-CM maturation strategies in recapitulating the *in vivo* changes in CM proteome during maturation. As a relevant example, Gorska et al. investigated mTORC1 specific gene expression changes in CM hypertrophy by performing ribosome-sequencing on neonatal rat ventricular cardiomyocytes (NRCMs) treated with phenylephrine ± Torin 1. Comparing the results in samples with/without Torin treatment reflected the mTOR-specific translation identifying Cand2 as an mTORC1-dependent inducer of pathological hypertrophy (73).

Despite the critical role of mTOR in CM growth and development (see Figure 3), the exact molecular mechanisms and transcriptional networks invoked by mTOR inhibition have not been determined. In this regard, there still is a lack of understanding on how mTORC1/C2 activity changes during CM differentiation and maturation. This is partly due to the disturbing and potentially lethal effects of mTOR genetic modulation in both *in vivo*/*in vitro* studies (74). Fortunately, recent technological advances can help one monitor real-time changes in mTOR signaling. Bouquier et al. developed an mTORC1 biosensor (AIMTOR) based on protein proximity associated resonance energy transfer (BRET) between a bioluminescent donor and a fluorescent acceptor as part of one biosensor which contains mTORC1 target peptide ULK1 (15). Previous mTORC1 biosensors have been reported using fluorescence RET (FRET) technology (14). However, one advantage of BRET biosensor is the lack of phototoxicity, photobleaching, and ∼10 times higher signal-to-noise ratio. The mTORC1 phosphorylation of ULK1-derived peptide causes a conformational change in biosensor enabling RET and measurement of subtle changes in mTORC1 signaling in hiPSC-CMs expressing the biosensor (unpublished work) which can also be directly visualized using BRET microscopy (16).

This approach can be especially informative by using different versions of the biosensor with different subcellular localization to monitor the change in mTORC1 signaling during CM differentiation in various subcellular compartments such as the nucleus. In fact, the nuclear presence and activity of mTOR have recently gained attention due to the advancements in genomics and biophysical technologies that made it possible to monitor the activity of mTOR. For instance, DEPTOR (DEP domain-containing mTOR interacting protein) has recently been shown to have transcriptional activity in the nucleus in addition to its role on inhibition of mTORC1/C2 signaling (16). Therefore, transcriptional regulation by mTOR is hypothesized to be via directly acting on chromatin as TF/co-factor besides its role in the regulation of epigenetic modifiers and TFs activity (17) which emphasize the potential of future research on monitoring mTOR activity in the nucleus and on transcriptional regulation in CM development and transition to adult state.

### Hormonal cues in cardiomyocyte maturation

Both thyroid hormone (TH) and DEX are critical factors mediating the neonatal adaptation to the extrauterine environment with a surge in activity/levels from the latest stages of gestion (TH: ∼36 weeks of gestation, DEX: ∼28 weeks of gestation) to postnatal life (75). During gestation, the metabolism of T4 to the active form (triiodothyronine or T3) and relatively inactive forms (rT3, sulphonated T3) is regulated by developmental-stage specific expression of deiodinase enzymes. The fetal plasma levels of T3 remain low during gestation. However, near term, the deiodination of T4 to active form T3 and its reduced clearance causes an increase in T3 level (76). In this regard, glucorticoid hormones are responsible for the conversion of T4 to active T3 form by a change in the expression of specific deiodinase enzymes such as Dio2 in fetal CMs. DEX, therefore, is used to enhance T4/T3 conversion as shown by studies in sheep model and as a common practice in antenatal care in preterm deliveries (76, 77).

According to a study by Wang et al., 2 weeks of treatment of hiPSC-CMs with 100 nM T3 and 1 μM DEX starting from day 16 of differentiation, enhance the electrophysiological maturation of hiPSC-CMs as evidenced by reduction in the spontaneous beating rate, shortening of the action potential duration, accelerating upstroke velocity, and generating a more negative resting membrane potential. This was shown to be the result of an increased expression of a variety of potassium channel encoding genes (i.e., *KCNJ2, KCNJ12, KCNH2, KCNE2, KCNQ1*) and decreased expression of the gene encoding the ‘pacemaking channel’ *HCN4* which is responsible for the funny current (I_*f*_). The functional analysis by patch clamp and optical mapping confirmed decreased funny current (I_*f*_) and increased potassium/sodium current (I_*k*1_, I_*Kr*_, I_*ks*_, I_*NA*_). Accordingly, the enhanced conduction velocity in treated hiPSC-CMs was also shown by enhanced expression of the gap junction protein connexin 43 mRNA and proteins levels (78).

This group previously reported another aspect of T3 and DEX treatment on CM maturation by evaluating of the ultrastructure and calcium signaling with the same dosage and duration of treatments. Using confocal imaging, Parikh et al., observed that a combination of Matrigel mattress and treatment with T3 and DEX is sufficient for developing t-tubules and widespread cellular distribution of essential t-tubule related proteins like Cav 1.2 (voltage-gated L-type calcium channel) and RyR2 (ryanodine receptor 2) organization (79). De La Mata et al. showed that the T-tubule associated protein BIN1 (Bridging integrator 1) expression promotes the formation of the extensive tubular distribution and suggested that its expression along the sarcolemma can promote clustering of Cav1.2, which increases the chance of cooperative gating and calcium influx involved in excitation contraction coupling. They also posited that the BIN1 stabilizes calcium release by functioning as anchoring proteins for junctional sarcoplasmic reticulum (SR) where RyR2s are located. Therefore, BIN1 has a significant role in PSCs maturation (80). The necessity of combined TH and DEX treatment aligns well with the reported interdependence of glucorticoid and TH signaling.

In accordance with the Wang et al. findings, Gilani et al. used a rat model of TH deficiency to show the effects of T3 on cardiac function. The results of this study revealed an association between TH deficiency, disorganized T tubules, and reduced RYR2 cluster size/number. TH treatment increased the expression of junctophilin2 (*JPH2)* as the critical regulator of cardiac dyad structure, and enhanced T-tubules network and RYR2 organization (81).

Additionally, Skorska et al. used super resolution microscopy and machine learning to quantify the effects of TH and DEX treatment on sarcomere maturation in hiPSC-CMs and they identified a significant enhancement in sarcomere length and orientation after 7 days of treatment starting from day 25 of CM differentiation. However, there was no significant change in the density of sarcomere network and the thickness of Z-disk (82).

In a recent study by Tsan et al. using RNA-seq analysis to compare hiPSC-CMs subjected to chemical versus mechanical maturation show that maturation of mitochondrial respiration is more reliant upon chemical maturation compared to mechanical cues. The transcriptome hallmarks of mitochondrial maturation were highly enriched in glucose-free RPMI (composed of B27, 4 mM galactose, 2 mM GlutaMAX (l-Alanyl-l-glutamine), 4 mM lactate, and 0.5 mM pyruvate) ± 100 nM T3 conditions (83). Moreover, mechanical cues were shown to be less effective compared to chemical cues in the improvement of TNNI3 and beta 1 adrenergic receptor (ADRB1) expression. Treatment with the above-mentioned media + T3 improved the adrenergic response and the switch from SSTNI to cTnI as demonstrated by the increased mRNA expression of *TNNI3* and its protein levels by mass spectrometry and immunofluorescence (84).

Notably, a promising study by Funakoshi et al., developed a protocol for induction and maturation of compact CM from hESC-CMs. The authors showed that 9 days of treatment compact ventricle CMs and atrial CMs from day 18 of differentiation with a maturation combo in media containing 11.1 mM glucose, 200 μM palmitate, 1 μM GW 7647 (a PPARa agonist), 254.8 nM DEX, and 4 nM T3 followed by five additional days of treatment with only 200 μM Palmitate (in low glucose media) significantly enhanced mitochondrial maturation, fatty acid β oxidation, well defined sarcomeres with discernible Z lines, I/A bands, calcium handling, and more mature transcriptome. ERRα (Estrogen-related receptor A) was further identified as one of key factors regulating CM maturation (85). Future electrophysiological analysis would be informative to evaluate the detailed effects of this maturation protocol on the ion channel functionality of these CMs. Furthermore, the engraftments of mature CMs into rat heart showed a promising profile compared to immature CMs. Still, impaired CX43 localization to cell junction was observed despite high CX43 expression (85). As previously discussed, it would be informative to evaluate whether combined mTOR modulation enhances this challenging aspect of CM maturation.

Wickramasinghe et al., recently identified the isoform-specific effects of PPAR signaling in CM maturation. PPARa, the major isoform expressed in PSC-CMs, is usually activated to enhance CM maturation by previous studies (15, 63, 85). In this study, the treatment with isoform specific PPAR agonists detected PPARd as the most critical in enhancing different aspects of CM maturation. The treatment of both hPSC-CMs 2D monolayers and engineered heart tissues (EHT; starting 2 weeks after replating of day 20 CMs for a duration of 4 weeks treatment) with 2.5 μM GSK0660 (a PPARδ agonist) enhanced fatty acid oxidation, sarcomere alignment, CM size, binucleation, electrophysiology (e.g., action potential duration), and contractility. Still, T-tubule formation, gap junction proteins expression/localization, and some aspects of electrophysiological maturation remains to be investigated in future research (25).

To investigate the role of steroid hormone signaling in CM maturation, Sim et al. performed single nucleus RNA-seq on left ventricular cardiac tissues of healthy human donors identifying sex-specific transcriptional profiles at different stages of cardiac development on a largely CM population. The subsequent bulk RNA-seq and ATAC-seq analysis on CM-specific nuclear marker (PCM1) enriched nuclei from 21 healthy left ventricle cardiac tissues (10 male, 11 female, from 14 weeks of gestation to 65 years old) detected a considerable number of genes with differential expression in a sex-specific manner and a major change in chromatin accessibility from fetal (19–20 weeks of gestation) to neonatal (up to 4 years of age) (83). The increased chromatin accessibility was shown to be enriched for binding sites of steroid hormone nuclear receptors such as progesterone receptor (PGR) and glucocorticoid response elements (mostly male CMs) and AP1-JUN TFs family (mostly female CMs) in CM maturation. The increased PGR expression during CM maturation (male and female CMs) was not detected when compared with mouse CMs potentially highlighting species differences in CM development (83). Overall, Sim et al. verified the maturation enhancing effects of progesterone (PG) on CMs contractility and electrophysiology by PG treatment of hESC-CMs and human cardiac organoids (HCO). Moreover, *in vivo* mouse expression of human PGR using Adenovirus-associated gene delivery, induced the transcription of metabolic maturation associated genes (83).

Miki et al. used TNNI1*^EmGFP^*/TNNI3*^mCherry^* double reporter hiPSC lines as a method to screen for compounds which enhance CM maturation (i.e., increased TNNI3*^mCherry^* signal). Treatment with an Estrogen-related receptor gamma (ERRγ) agonist from day 8 until day 16 of differentiation was shown to enhance numerous aspects of CM maturation including detectable sarcomere M lines, increased sarcomere length, cell area, mitochondrial/contractile maturation, action potential amplitude, maximum upstroke velocity, and conduction velocity. Notable to this study, T-Tubule formation, a major hallmark of CM maturation, was observed in ERRγ agonist treated hiPSC-CMs in the absence of other maturation enhancing strategies (79). The result of this study is in accordance with Sakamoto et al. findings on role of ERRα and γ Signaling in normal CM maturation in mice and hiPSC-CMs structural and functional (e.g., electrophysiology and metabolism) development (80). Moreover, ERRα was also detected to play a key role in structural and metabolic maturation of hiPSC-CMs in the study led by Funakoshi et al. (85). Sakamoto et al. further showed that the transcriptional regulation of CM maturation by ERRγ is mediated in a GATA-4 cooperative manner for cardiac ion channels and contractile apparatus. However, the regulation of mitochondrial metabolism was shown to be independent from GATA-4 activity. Moreover, PGC-1α activity was recognized for its essential role in the transcriptional regulation by ERRγ (86).

### Other signaling pathways

Other signaling pathways have also been shown to be involved in CM maturation (for an overview of pathways potentially involved in CM maturation refer to Figure 1). For instance, Ho et al took advantage of the shift from glycolysis to fatty acid oxidation as a selection marker for mature CM s. They performed RNAseq analysis on hexokinase 1-GFP tagged hiPSCs-CMs sorted based on hexokinase expression from fetal (high GFP) and to mature (low GFP) states. The results of this study showed the upregulation of neutrophile degranulation and interferon signaling in more mature CMs. Subsequently, treatment of hiPSCs-CMs from day 7 of differentiation with 25 ng/mL (i.e., 1.5 nM) IFN-γ for up to 10 days showed an enhancement in the expression of genes encoding ion channels (*KCNJ2, SCN3B, SCN5A*), *MYH7* to *MYH6* ratio, and fatty acid β oxidation among others. Moreover, increased sarcomere length, Ca^2+^ dynamics, and electrophysiological maturation (e.g., upstroke velocity, beat period) were also observed (87).

In accordance with this study, a recent investigation using single nucleus RNA sequencing on the left ventricle of human cardiac tissues of healthy donors from different ages (fetal, young, and adult) also identified increased interferon alpha-beta signaling/response during cardiac maturation, albeit in non- CM population of cardiac tissue such as endothelial cells, immune cells, and neurons among others (83). The pro-maturation effects of IFN-γ in the Ho et al. study were shown to be via upregulation of the JAK-STAT signaling pathway (87). JAK-STAT signaling is involved in the regulation of inflammatory responses among others. The modulation of inflammatory signaling is one aspect of CM maturation that is understudied. In fact, the lack of immune cells incorporation is one of the shortcomings of current multicellular heart models (88). The results of previous studies indicate the critical role of macrophages (less than 7% of heart cell population) on heart through enhanced electrophysiological conduction (88, 89).

The activity of immune modulatory factors such as the Nuclear factor kappa B (NF-κB) is reported to be essential for CM maturation through binding to the promoters of CM maturation genes and increasing their expression. Pharmacological inhibition of NFκB thereby was suggested to suppress CM maturation; though, the role of NF-κB needs to be evaluated in terms of the functional aspects of CM maturation (90). On the other hand, the Nuclear factor-erythroid factor 2-related factor 2 (Nrf2) which negatively regulates NF-κB and JAK-STAT signaling pathway, is also reported to be critical for CM metabolic and structural maturation (91, 92). One aspect of the Nrf2 role in CM maturation is by enhanced mitochondrial respiration (91). Mitochondrial respiration increases ROS which activates JAK-STAT signaling. In addition, the upregulated ROS levels increase Nrf2 activity as a TF to counteract oxidative stress by inducing the expression of antioxidant genes. It remains to be studied how the activity of these different inflammatory factors with feedback loops are regulated during the time-course of CM maturation.

According to a recent study, NrF2 encoded by the *NFE2L2* gene is highly expressed during the early stages of mouse *in vivo* CM differentiation which aligns well with its role in enhancing mitochondrial respiration as part of early CM metabolic maturation. However, the time-course of *NFE2L2* activity *in vivo* is not recapitulated in *in vitro* PSC-CM differentiation and was posited to be one of the key factors responsible for maturation arrest of *in vitro* CM differentiation (93). It is complicated to interpret the significance of NFE2L2 stage-specific activity in CM maturation; however, increased ROS levels is an important inducer of DNA damage response which is implicated in CM proliferation arrest as part of CM maturation process (94). DNA damage response, in fact, is shown to be responsible for neonatal mouse CM proliferation arrest upon transitioning to oxygen-rich environment at birth. It is further shown that ROS scavengers delay cell-cycle arrest (13). Therefore, due to the role of NrF2 on modulating DNA damage response, it may be one of the reasons that its activity and expression should be fine-tuned during the progression of CM maturation. Moreover, it could be also argued that dysregulated Nrf2 activity may be a hallmark of abnormal conditions in CM maturation protocols that interferes with normal progression of CMs to a matured state.

### Thermogenesis as a potential stimulus in cardiomyocyte maturation

Finally, an exciting link between endothermy in newborn mice and the loss of CM regenerative potential mediated by TH signaling was recently identified. It is postulated the increased metabolic rate upon birth, due to the adaptive thermogenesis, interferes with CM proliferation further causing cell cycle arrest. The interaction between sympathetic nerves adrenergic receptors as the regulators of thermogenesis and TH is opined to be responsible for this phenomenon as shown by the positive effect of combined inhibition of TH and adrenergic signaling on CM proliferation (95). Therefore, the link between mammalian thermogenesis and loss of CM proliferation as part of CM maturation process seems a promising unexplored area in the field of CM developmental biology.

In this regard, human newborns in the first six months of life are relatively incapable of thermoregulation through muscle shivering thermogenesis and rely on non-shivering thermogenesis enabled by the presence of fat deposits known as brown adipose tissue (BAT). This type of adipose tissue dissipates energy in the form of heat and is an attractive area of research due to its detection in adult humans and its possible role in health and disease (96). The activity of BAT and non-shivering thermogenesis is dependent on TH signaling (97). According to recent research, human breast milk is enriched of a brown fat activating lipokine 12,13-diHOME which is shown to have protective effects on cardiac hemodynamic and mitochondrial respiration in mice CM s (98–100). The process of human CM maturation is argued to be in progress for several years after birth (101) and it would be informative to investigate whether BAT secretome and postnatal thermogenesis regulation modulate the transition of fetal CMs to adult state.

## The impact of preterm birth on cardiomyocyte maturation

The transition from a prenatal to postnatal environment at birth is believed to be a critical step in CM development and transition to an adult state. One aspect that is worthy of consideration is how this process is affected in preterm birth scenarios (with a global prevalence of more than 10% of live births) and how an unprepared heart adapts to the sudden unexpected environmental change and interruption of a critical phase in CM maturation (102). This is of interest since the transcriptomic analysis of hiPSC-CMs indicate the limitation of current CM differentiation strategies in which CM s resemble the first trimester fetal heart which further may progress to around the equivalent of a second trimester fetal heart upon maturation treatment (103). The results of other studies also suggest a maturation arrest in hiPSC-CMs not improving beyond late embryonic stage (16, 104, 105).

The normal term-born is defined as time of delivery ≥ 37 weeks of gestation. Preterm-born is categorized as moderate to late (32–<37weeks of gestation), very preterm-born (28–≤32 weeks of gestation), and extremely preterm-born (<28 weeks) (102, 106).

To illustrate the level of CM maturity at different stages of gestation, we refer to studies performed on human fetal heart using electron microscopy among other techniques. From this work, fetal CM s around 18–24 weeks of gestation exhibited minimally organized myofibrils at the periphery of CMs with underdeveloped thick and thin filaments and no detectable sarcomere A-bands, I-bands, and M-lines. By 32 weeks of gestation, the myofibrillar organization appears more enhanced but the M-line and H-band are still indiscernible. This is expected since the appearance of M-bands is considered as the hallmark of final myofibril maturation (107). Importantly, the T-tubules appear around this stage and IDs develop a more adult-like structure (i.e., more advanced desmosomes, fascia adherents, and gap junctions). Mitochondria at this stage of development appear round shaped with random cellular distribution at early weeks but localize to the periphery of nucleus and myofibrils by week 32 of gestation with increased number and variation in morphology. Elongated nuclei are observable at later stages around week 25 of gestation. Finally, binucleated CM s are observed from 32 weeks of gestation (75, 107). Interestingly, CM maturation continues for several years after birth as shown by previous research. According to Vreeker et al., the mechanical junctions (N cadherins, Zonula occludens-1, and desmosome proteins) are established at the ID by 1 year of age. However, the final localization of cardiac sodium channels (Na_*V*_1.5) at the ID and lateral side of CMs happen around age 2. The final localization of gap junction connexin 43 proteins at the ID are observed by 7 years of age (108). In agreement with this observation of *in vivo* CM development, current *in vitro* CM maturation strategies often fail to properly address the lack of CX43 localization at IDs despite the increased protein and mRNA expression levels. Therefore, future research needs to be performed to understand the underlying transcriptional regulation and post-transcriptional mechanisms which modulate the CX43 trafficking and localization at IDs after birth.

As mentioned above, the preterm heart is clearly underdeveloped in terms of ultrastructure and function. Moreover, thermoregulation (due to lack of sufficient BAT deposits and response to cold) and other physiological aspects of transitioning from intrauterine to extrauterine environments are disrupted in preterm infants (109). While preterm birth was previously associated with high mortality, advancements in science and clinical practice have significantly increased the survival rate to adulthood. This has further been accompanied by a surge in studies that indicate the long-term cardiac abnormalities in preterm-born individuals including decreased ventricular chamber size, increased ventricular wall thickness, arrhythmias, myocardial fibrosis, and reduced cardiac output due to impaired cardiac cycle (102, 106, 110–112). Hence, the knowledge on the molecular mechanisms underlying the accelerated adaptation or maladaptation of developmentally underdeveloped CM s to extrauterine environment in comparison to normal developed CM s may be useful to gain insight on improving *in vitro* maturation strategies. While it is challenging to perform basic mechanistic research (e.g., ethical aspects and variable confounding factors), previous research has been performed on deceased preterm, still-born, and elective aborted fetuses (83, 113). Furthermore, animal models of preterm birth such as sheep are useful to study CM maturation in the context of preterm birth because of similarities to human perinatal CM growth (114, 115).

Another potential advantage of research on the preterm heart is to better recognize the possible long-term effects of current maturation treatments on hiPSC-CMs used for cardiovascular regenerative therapy. For instance, some adverse effects such as CM hypertrophy and decreased CM number have been reported from postnatal glucorticoid therapy (e.g., DEX) in preterm infants (115). Moreover, some studies indicate the inhibitory effects of exogenous glucorticoid treatment on normal heart maturation during specific gestational timeframes (116, 117). Based on a study by Ivy et al., antenatal glucorticoid treatment at mid/late gestation, decreases the CM expression of glucorticoid receptor (GR) and its target gene PPAR gamma coactivator 1-alpha (PGC1A) in sheep model. PGC1A is the master regulator of CM energy metabolism and a critical mediator of the pro-maturation effects of glucorticoid. Therefore, the mistiming glucorticoid treatment may interfere with normal CM maturation (77). As suggested by Ivy et al., the impact of T3 and DEX on maturation may be due to the interdependence of TH and DEX in which TH should be at an appropriate level to observe a pro-maturation effect from glucorticoid treatment (77, 118). Another study, however, showed enhanced CM maturation by DEX-only treatment in mouse ESC-CMs. According to the authors, mistiming of glucorticoid treatment and excessive dosage induces pathological hypertrophy instead of physiological hypertrophy in late fetal gestation. Moreover, excessive dosage may induce uncontrolled mitophagy which further affects normal mitochondrial function and CM health. It should be noted that *in vitro* PSC-CMs lack endogenous glucorticoid supply compared to *in vivo* models. Therefore, this could be one of the reasons for different results observed in *in vivo* heart models compared to *in vitro* CMs in addition to other whole-organ related effects overlooked in *in vitro* studies (42). Overall, glucorticoid treatment for *in vitro* CM maturation and antenatal care applications should be optimized with respect to dosage, developmental timing of treatment, and the possible connection with TH levels among other factors.

## Role of matrix physical properties in human induced pluripotent stem cell-derived cardiomyocytes maturation

The remodeling of the ECM and the interaction between ECM and CM s are critical regulators of CM maturation during heart development. Chanthra et al., used a mouse ESC line with an RFP labeled sarcomere M-band protein (Myom2-RFP) to identify CM maturation enhancing stimuli which correlated with increased expression of as a major hallmark of mature CM. Laminin-511/521 was further detected to be the better ECM choice for improving mouse CM maturation as verified by different functional analyses (119). The change in the ratio of ECM proteins such as collagens is accompanied by a significant change in stiffness of CM environment from mesoderm (∼ 500 Pa) to adult heart tissue (∼10 kPa) (120). The increased stiffness beyond physiological levels is observed in pathological conditions such as hypertrophic cardiomyopathy and heart failure (121) which are accompanied by transitioning of CMs back to an immature state as previously discussed (50, 51). The current use of rigid cell culture surfaces is among the culprits thought to interfere with the recapitulation of normal *in vivo* CM maturation. For instance, previous research indicated that higher substrate stiffness (∼1,000 kPa) to be the most desirable for survival and proliferation of mouse fibroblasts with a ∼45% decrease in fibroblast survival rate by reduced stiffness to ∼280 kPa (122). Respectively, fibroblasts are observed as part of non- CM population in hiPSC-CMs differentiation protocols; it will be interesting to evaluate whether substrate stiffness can be optimized to improve the CM differentiation efficiency and the ratio of non- CM population in a manner that better improves CM maturation. In fact, previous research using different ratios of hiPSC-CM/-non-CM population co-culture emphasizes the importance of a balanced non- CM population contributing to hiPSC-CMs maturation (123, 124). While a higher ratio of non-CM population interfered with electrophysiological maturation, an optimal purity level of 70–90% CMs was shown to be the most favorable for improving the maturation profile (123). Accordingly, Giacomelli et al. developed three-dimensional cardiac microtissues by incorporating of 70% hiPSC-CMs, 15% hiPSC-cardiac fibroblasts, and 15% hiPSC-endothelial cells showing enhanced maturation profile in various structural and functional aspects including T-Tubule formation with an upregulation in beta-adrenergic signaling and CX43 mediated crosstalk contributing to maturation enhancing effects of multicellular tissue with special impact from cardiac fibroblasts (125). A transient maturation-promoting effect from cardiac fibroblasts populations in hiPSC-cardiac microtissues was also noticed in a recent study by Hookway et al. (126). In this regard, the role of cardiac fibroblast populations in mouse postnatal CM maturation was recently investigated. Hortells et al. identified a decrease in cardiac ECM fibronectin levels in transitioning from neonatal to adult stage with upregulation of fibrillar collagens (in particular types I and III) by postnatal day 30 (P30) with a peak of expression at P7 corresponding to the timing of CM proliferation arrest and induction of hypertrophic growth (127). The authors further reported a population of periostin (Postn)-expressing cardiac fibroblasts from postnatal day 0–11 whose ablation significantly interfered with CM maturation through inhibition of hypertrophic growth, CM binucleation, dominant fetal TNNI1 expression, and increased cardiac sympathetic nerve area manifested with a deeper Q wave in electrocardiogram (ECG) analysis (127). In accordance with the transient presence of pro-maturation fibroblasts populations in mouse neonatal heart, Cho et al observed the transition of hPSC-CMs from immature to adult stage only when transplanted into neonatal rat hearts (128). These findings highlight the exciting notion that stage specific stimuli present in neonatal heart may contribute to transition of fetal heart to adult stage which remain to be investigated in future research (128).

The relationship between cardiac Postn-fibroblasts, sympathetic innervation, and CM maturation is not well understood. However, Kowalski et al recently investigated the role of sympathetic neurons in CM maturation. On day 10 hiPSC-CMs were co-cultured with sympathetic neurons obtained from sympathetic ganglia of late gestation mouse embryos (E13.5). The subsequent analysis after 30 days of co-culture revealed a significant enhancement in sarcomere alignment and the expression of maturation genes such as *KCNH2, KCNQ1, KCNJ2, SCN5A*, and *RYR2* to name but a few. Interestingly, CX43 expression, not ID localization, was shown to be enhanced by immunostaining despite unchanged mRNA expression highlighting the modulation of CX43 beyond transcription level (129). Moreover, the change in CX43 levels aligned well with Giacomelli et al. findings on cardiac microtissues previously mentioned (125, 129). Despite observing some maturation-promoting effects, sympathetic neurons co-culture were not quite optimal regarding sarcomere length and some aspects of calcium handling maturation (129).

Another major focus of current research is to better mimic the change of ECM to recapitulate normal CM maturation environment (104). Young et al. observed a decrease in ECM proteins fibronectin and laminin mRNA expression, increased collagen from ∼11 days post-fertilization, and myocardial stiffness to ∼ 8.2 kPa after ∼17 days post-fertilization in embryonic chicken heart. They further used collagen-coated hyaluronic acid hydrogels with dynamic stiffening over time similar to the change in stiffness of chicken heart to evaluate the impact on embryonic chicken CM maturation. The results of this study showed an enhancement in the expression of cardiac troponin T, myofibril length and orientation compared to the less stiff polyacrylamide hydrogels (130). Herron et al., compared the effects of different ECM conditions on hiPSC-CM maturation showing the combination of polydimethylsiloxane (PDMS) (40D, tensile strength of ∼1000 kPa) + Matrigel as the softest ECM to be the most effective as reflected by the enhanced expression and localization of maturation markers such as CX43 and electrophysiological maturation parameters as compared to ECM coated rigid glass coverslip (∼GPa) and PDMS + fibronectin (131). Dhahri et al., cultured hiPSC-CMs on PDMS-lined roller bottles generating CMs with enhanced electrophysiological, structural, and functional maturation (132). This includes improved myofilament alignment, Cx43 expression, sarcomere length, and improved integration into host cardiac tissue.

Chin et al., studied polyacrylamide hydrogels with a stiffness range of 2–33 kPa, coated with different ECM components, on CM morphological characteristics and mechanotransduction. H9C2 cells and neonatal rat CM s (NRCMs) were cultured on coated substrates for ∼4 days and subsequent functional analysis showed the correlation between increased stiffness and cell size (with a plateau phase ∼12 kPa), nucleus size, and stiffness of single cells as shown by single cell AFM in H9C2 cells (120). However, a significant observation was not shown in NRCMs. Moreover, no correlation was detected between substrate stiffness and sarcomere length. The immunofluorescent staining showed an increased nuclear localization of key mechanosensitive proteins with respect to the coating used. For instance, in H9C2 cells, Yap nuclear localization in response to increased stiffness was only observed in collagen/laminin but not fibronectin coated polyacrylamide hydrogels (120). Previous research shows the pivotal role of ECM proteoglycan Agrin in neonatal mouse CM in the sensing of stiffness signals and activating Yap nuclear localization by negative regulation of Hippo pathway. Agrin is shown to be replaced by another ECM protein during mouse CM maturation inactivating Yap nuclear localization and CM proliferative potential (133, 134). The association between physiological ECM stiffness and enhanced cardiac gene expression in direct CM reprogramming (i.e., iCMs) was also shown to be in part through suppressed Yap/TAZ signaling (135). Though, other studies argue that contractility induced nuclear deformation, not substrate stiffness, is responsible for Yap nuclear transport (136). Due to the essential role of Yap transcription coactivator in modulating different aspects of CM maturation such as cell proliferation and hypertrophic response, it would be instructive to further evaluate the dynamics of its activity at different stages of PSC-CMs differentiation and in response to different maturation strategies.

**FIGURE 4:**
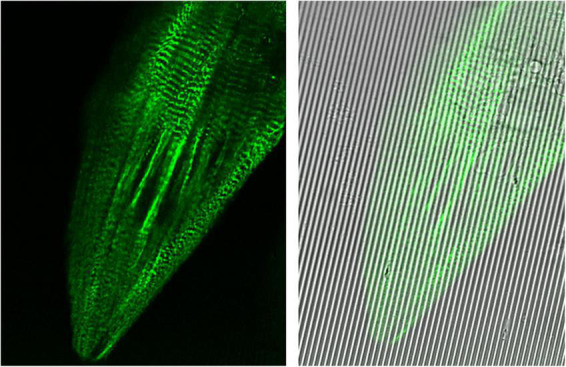
Images captured at 100× oil immersion objective using Leica Sp8 Confocal microscope. hiPSC-derived cardiomyocytes (CMs) expressing mEGFP-labeled α-actinin-2 at sarcomeric z-disks (cell line ID: AICS-0075 cl.85) replated on Curi Bio nanopatterned substrate at day 15 of differentiation and treated with metabolic maturation media for 2 weeks.

The recapitulation of cardiac ECM topography is another aspect of improving CM maturation which has been previously linked with enhanced CM and myofibrillar alignment among others (see Figure 4). Knight et al., used the combination of plating hiPSC-CMs at day 35–40 of differentiation on 20 μm micropatterned grooves and treatment with 50 μM palmitic acid, 100 μM oleic acid, and 10 mM galactose. Functional analysis on day 65–75 of differentiation, showed a significant enhancement in CM morphology, myofibrils contractile force generation, response to adrenergic signaling (phenylephrine: selective α1-adrenergic receptor agonist), and enrichment of fatty acid metabolism gene programs. However, electrophysiological and metabolic assessments were not performed (137).

In another study, day 15 hESC- CMs were plated on substrates with microgrooves of variable widths (5, 10, 15 μm). Subsequently, CMs were treated for 8 days with 100 nM T3 and 5 days simultaneous electrical pacing (i.e., starting at 0.2 Hz at day 3, 0.5 Hz at day 4, and 1 Hz at day 5-8). The optical mapping analysis further identified the highest conduction velocity in T3 + electrical paced CMs cultured on substrates with grooves of 15 μm. Moreover, T3 + electrical paced CMs cultured on substrate showed faster maximum upstroke velocity and increased *I*_*Na*_, lower automaticity as confirmed by reduced *I*_*f*_ and *HCN2/HCN4* expression, enhanced calcium handling demonstrated by gene expression and optical mapping calcium transients. RNA-seq analysis identified TGFB signaling as the key pathway downregulated in combinatorial treatments (138).

Tsan et al. recently developed a two-dimensional cardiac muscle bundles (2D MBs) platform (84) as an improvement to their previous study on single micropatterned iPSC-CMs (139). The day 16 metabolic purified hiPSC-CMs were re-plated to stiff PDMS with growth factor reduced Matrigel. Subsequently, hiPSC-CMs at day 24–25 of differentiation were re-plated to the micropatterned 8 kPa PDMS substrates with fibronectin. The imaging analysis of GFP-tagged CMs (Allen Institute hiPSC lines), showed a significant improvement in myofibrillar alignment, resting sarcomere length (∼2.2 μm), and contractile function. Moreover, a significant enhancement in CX43 expression and proper localization was shown after 10 days 2DMBs formation (84). Other aspects of CM maturation were markedly improved as shown by functional assays (patch clamp, calcium handling) and more mature transcriptome (e.g., *HOPX, SCN5A, KCNJ2, RYR2*) as compared to chemical maturation strategies. One notable aspect of this study is the use of Mavacamten (500 nM, contractility inhibitor) to show the crucial role of contractile activity in myofilament development. Another interesting aspect is the illustration of the critical role of physiological extracellular calcium concentration (1.45 mM instead of 0.42 mM used in RPMI1640 media) in the development of more mature calcium handling. T-tubule formation, however, was not mentioned or evaluated in this study (84).

## Engineered heart tissue

Human EHT and 3D cardiac models are critical advancements in the field of cardiovascular regenerative medicine accelerating the progress in improving CM maturation and disease modeling. Zhao et al., developed a ventricular and atrial Biowire platform by seeding a combination of hiPSC-VCMs/ACMs and cardiac fibroblasts at a ratio of ∼10:1, 1.5 (in collagen hydrogel matrix) on polystyrene microwells attached to polymer wires (POMaC) for progressive electrical conditioning (2-6 Hz). Moreover, the composition of new (i.e., Biowire II compared to Biowire I (140)) platform was suggested to eliminate the issue with PDMS absorption of hydrophobic drugs in pharmacological investigations (141). Electrical conditioning in the Biowire II platform further enhanced sarcomere organization and chamber specific transcriptomic hallmarks of structural and functional maturation. Moreover, more adult-like chamber specific Ca^2+^ handling and electrophysiology such as force-frequency relationship (FFR), post-rest potentiation, and conduction velocity were observed (141).

Lu et al subjected ring-shaped EHT (calamari model) from hiPSC-CMs in collagen hydrogel matrix to daily 1 Hz electrical stimulation, 0.1 nM T3 treatment, and progressive stretch (up to 0.32 mm/day) for 3 weeks. The stretch conditioned EHTs demonstrated many aspects of more mature CM such as increased sarcomere length, myofibrillar alignment, enhanced calcium handling/positive FFR, electrophysiological maturation (e.g., greater resting membrane potential and upstroke velocity), and adrenergic response among others (142). Still, T-tubule formation and localization of CX43 proteins at IDs were not observed (142).

Ronaldson-Bouchard et al., generated EHTs using a combination of fibroblasts and hiPSC-CMs at the onset of beating in fibrin matrix. They further subjected the early stage CMs to static stretch and constant versus progressive electrical stimulation. The electromechanical conditioning for 2 weeks including progressive electrical stimulation of 0.33 Hz/day (from 2-6 Hz) followed by a subsequent one week of stimulation at 2 Hz, showed the best maturation enhancing effects with respect to numerous parameters notably T-Tubule formation, positive FFR, pronounced adrenergic β receptor response, and more mature transcriptomic/metabolic/electrophysiological profiles. Importantly, these favorable effects were achieved only when performed at an early stage time window of CM differentiation (143).

Earlier timepoint electrical stimulation from day 0 of differentiation until day 15 for 2 h daily pacing at 1 Hz has been shown by other studies to enhance the differentiation of hiPSCs toward cardiac conduction-like cells as determined by specific electrophysiological characteristics and enhanced expression of IRX3 which is linked with maturation of mouse ventricular conduction system (i.e., His-bundle and Purkinje fiber network (144)) and higher connexins 40 versus 43 expression among others (145).

Pretorius et al. cultured engineered cardiac-muscle patches (> 2.1 mm thickness) from hiPSC-CMs in fibrin matrix and performed electromechanical conditioning for 10 days (i.e., static stretch + 2 Hz electrical stimulation of 15 or 22 V). The results of this study indicated a significant enhancement in structural aspects of CM maturation as shown by TEM and enhanced mRNA/protein levels of maturation genes in cardiac tissues subjected to a combination of static stretch + 20 V electrical stimulation (146).

Huebsch et al. used the combination of Heart-on-chip platform composed of 80% hiPSC-CMs + 20% hiPSC-stromal cells and 10 days of MM (100 μM palmitic acid, 125 μM oleic acid, 2.8 mM glucose, 10 mM galactose, and 2.5% Bovine Serum albumin (BSA, including the amount in B-27 supplement) treatment. The results of this study also indicated an improved mitochondrial structure, oxygen consumption rate, electrophysiological aspects (e.g., action potential duration), and Ca^2+^ handling. However, the decreased mRNA expression of *KCNJ2* and increased *HCN2* were observed upon treatment with maturation media which are not consistent with a maturing phenotype. Moreover, the formation of T-tubules was not detected (147).

Finally, hCO or cardioids that are composed of the major cardiac cell types in a spatially organized manner, may provide a better understanding of cardiac tissue morphogenesis and disease pathogenesis (148–150). In this regard, the transcriptomic analysis comparing hiPSC-CMs 3D EHTs, 2D monolayers, and hCO with both human fetal and adult myocardial CMs determined hCO to be the best model in terms of recapitulating the transcriptome of the adult myocardium. Importantly, the results of this study indicate the enrichment of immune regulatory pathways in adult myocardium that is not recapitulated in *in vitro* CM models (observed to a minor extent in hCO) (151). This finding is relevant to what was reported by Sim et al., Ho et al., and Fang et al. among others (37, 83, 87–89). This further emphasizes the need for immune cell incorporation in multicellular heart tissues as previously mentioned (151). The impact of organoid models on CM maturation was recently shown by Silva et al. (152); the authors generated hiPSC-derived multilineage organoids [i.e., cardiac (mesoderm) and gut (endoderm)] with pronounced structural and functional maturation effects especially on atrial/nodal CMs. Moreover, the media used for organoid culture which included more physiological relevant extracellular calcium concentrations and 100 μg/mL ascorbic acid improved the maturation of hiPSC-CMs 2D monolayers as well similar to the findings of Tsan et al. and Feyen et al. using MM (0.5 mM ascorbic acid) (24, 84, 152).

## Transcriptional regulation of human induced pluripotent stem cell-derived cardiomyocytes maturation

During CM differentiation, distinct gene programs are activated or repressed by the activity of key TFs that regulate cell type and developmental stages (153). Recent advances in monitoring chromatin accessibility using imaging-based methods and TF-DNA interaction profiling have made it possible to understand the transcriptional regulation in more detail (154). The maturation strategies with a focus on transcriptional regulation has been previously investigated. For instance, Veerman et al. reported the impact of prolonged hiPSC-CMs culture (i.e., 66 days in this study) on SCN5A isoform switch to the adult isoform as a prerequisite to study an inherited arrythmias caused by SCN5A mutation. The adult isoform of SCN5A (containing exon 6 vs. fetal isoform 6a) generated by alternative splicing, manifested the effects of a mutation located in exon 6 highlighting the importance of CM maturation in enhancing the degree of which PSC-CMs can be reliably used for disease modeling (155). The molecular mechanisms by which the prolonged culture leads to the observed isoform switch should be investigated in greater detail.

In an innovative study by Biermann et al., employed polyinosinic-polycytidylic acid (pIC), an epigenetic modulator commonly used in cell reprogramming studies, from day 3 to day 5 of differentiation corresponding to an early cardiac progenitor cell (CPC) state (KDR+/PDGFR+). The treatment of CPCs led to increased expression of proteins related to mitochondrial maturation, higher ratio of adult cTnT (i.e., isoforms 6 and 11), better contractile and electrical maturation, and higher oxygen consumption rate (152). While pIC treated CMs doubled in size, the rod adult shape morphology was only obtained when CMs were re-plated to 20-μm grooved soft PDMS substrates with ∼10 kPa stiffness (physiological range). RNA-seq and chIP-qPCR analyses identified the upregulation of Notch signaling and its ligand JAG1, and increased binding of H3K9ac (histone acetylation ∼active marker) on JAG1 and sarcomeric genes promoters. The maturation promoting effects of pIC was eliminated in the DAPT (NOTCH inhibitor) treated CMs, highlighting the essential role of Notch signaling in CM maturation (156).

Miklas et al. used a microRNAs-based approach (MiMac) to enhance hiPSC-CMs maturation through lentiviral CRISPR/CAS9 mediated genome editing at day 15 of differentiation to knock out miR-200a/miR-122, and to overexpress let-7/miR-452 as determined by detailed functional analysis. MiMac treated CMs showed enhanced fatty acid oxidation, cell area, and force of contraction. Moreover, HOPX which was previously linked with CM maturation (157) was upregulated being a shared target of MiMac components. The pro-maturation effects of HOPX were further shown to be through SRF mediated regulation of cell cycle activity (4). For a detailed overview on the role non-coding RNAs with a focus on long non-coding RNAs (LncRNAs) in CM maturation refer to (158).

The results of recent studies suggest that current maturation protocols produce CMs that are still far from the adult CM state; this failure is partly due to the fact that none of the methods are sufficient to fully activate all major TFs involved in CM maturation (93). Another major limitation of current methodologies is the absence of a global metric for the assessment of the degree of CM maturation reported by different studies. In this regard, Kannan et al. developed an entropy score-based method from single cell RNA-sequencing data to evaluate maturation in a quantitative manner at single cell level (105).

Future studies are needed to identify all the major transcriptional regulators involved in *in vivo* human CM maturation and how the dynamics of the activity of these TFs, the simultaneous activity of complex TF networks, and their direct target genes are modulated during the time-course of CM differentiation and maturation.

## Conclusion

The recapitulation of structural and functional characteristics of human adult cells is a major aim in many fields dealing with hPSCs including cardiovascular regenerative medicine. Here, the most recent studies were highlighted as an overview of maturation inducing protocols and the possible underlying mechanisms. Hopefully, recent technological advances, the knowledge of biochemical and physical MIFs, and defining a global metrics to evaluate the degree of CM maturation, will help to optimize the process of directing hiPSC-CMs to a fully matured state.

## Data availability statement

The raw data supporting the conclusions of this article will be made available by the authors, without undue reservation.

## Author contributions

HH: data collection, data analysis, and primary writer. PA, FJ, AA, and TB: data collection, data analysis, and writing. TS, EM, and WW: specific expertise editing. ST: overall editing. GT: concept developer, holder of funding, and writing. All authors contributed to the article and approved the submitted version.
